# Prognostic role of bioelectrical impedance phase angle for critically ill patients: A systemic review and meta-analysis

**DOI:** 10.3389/fmed.2022.1059747

**Published:** 2023-01-09

**Authors:** Wen-He Zheng, Yi-He Zhao, Yan Yao, Hui-Bin Huang

**Affiliations:** ^1^Department of Critical Care Medicine, Rehabilitation Hospital Affiliated to Fujian University of Traditional Chinese Medicine, Fuzhou, China; ^2^Department of Critical Care Medicine, Beijing Tsinghua Changgung Hospital, School of Clinical Medicine, Tsinghua University, Beijing, China

**Keywords:** critically ill, mortality, meta-analysis, prognosis, bioelectrical impedance phase angle

## Abstract

**Objective:**

Bioelectrical impedance-derived phase angle (PA) has exhibited good prognostic values in several non-critical illnesses. However, its predictive value for critically ill patients remains unclear. Thus, we aimed to perform a systematic review and meta-analysis to investigate the relationship between PA and survival in such a patient population.

**Materials and methods:**

We searched for relevant studies in PubMed, Embase, and the Cochrane database up to Jan 20, 2022. Meta-analyses were performed to determine the association between the baseline PA after admission with survival. We further conducted subgroup analyses and sensitivity analyses to explore the sources of heterogeneity.

**Results:**

We included 20 studies with 3,770 patients. Patients with low PA were associated with a significantly higher mortality risk than those with normal PA (OR 2.45, 95% CI 1.97–3.05, *P* < 0.00001). Compared to survivors, non-survivors had lower PA values (MD 0.82°, 95% CI 0.66–0.98; *P* < 0.00001). Similar results were also found when pooling studies reported regression analyses of PA as continuous (OR = 0.64; 95% CI 0.52–0.79, *P* < 0.00001) or categorical variable (OR = 2.42; 95% CI 1.76–3.34; *P* < 0.00001). These results were further confirmed in subgroup analyses and sensitivity analyses.

**Conclusion:**

Our results indicated that PA may be an important prognostic factor of survival in critically ill patients and can nicely complement the deficiencies of other severity scoring systems in the ICU setting.

## Introduction

Predicting prognosis in critically ill patients has always been a hot spot in critical areas. At present, some severity scoring systems have been established and widely used in critical practice, such as Acute Physiology and Chronic Assessment II (APACHE II), Simplified Acute Physiology Score II, and Sequential Organ Failure Assessment (1). These scoring systems use various vital signs, laboratory parameters, and imaging data. However, the scoring systems often lack accuracy and are overcomplicated due to many projects included (1, 2).

Bioimpedance analysis (BIA) is a non-invasive technology to measure the body’s electrical impedance at alternating current frequencies (3). Since the resistance and capacitive reactance characteristics are closely related to the human body composition, including muscle, fat content, and water content, it has been widely used in clinical analysis of body composition and capacity state assessment under stable conditions (4). However, BIA proved to be inaccurate in critically ill patients, leading to a significant overestimation of changes in total body water (TBW) (5). This is related to electrolyte transfer between and outside cells, and changes in fluid distribution, which commonly happen to ICU patients, can interfere with the BIA results (5, 6). Likewise, BIA results may be overestimated when extracellular water expansion occurs (i.e., heart failure, renal failure, or severe disease) (6, 7).

Interestingly, some components of BIA, such as phase angle (PA), a measure derived directly from resistance and reactance measurements, can be interpreted as an indicator of membrane integrity and water distribution between intracellular and extracellular spaces (8). A low PA reflects no fat mass loss and cellular dysfunction, while higher values (> 6 in normal subjects) reflect good cellular health or nutritional status. Theoretically, extracellular fluid composition, cell number, and membrane integrity are also closely related to disease severity, so it is also possible to use PA to predict disease severity and prognosis. PA has also been successfully used to indicate nutritional status and prognosis in patients with tumors, chronic kidney disease, and liver cirrhosis (9–12). However, several studies focus on PA in critically ill patients, and the conclusions are inconsistent (13–16). The different results might be related to the small sample size and the heterogeneity of the population among these studies. On the other hand, PA does not require parameters recall, body weights, and laboratory tests and has the advantages of simplicity, repeatability, and instantaneity (4, 16, 17). Thus, if PA can accurately reflect prognosis, it will nicely complement the deficiencies of other severity scoring systems.

Several studies have recently been published to investigate the association of PA with prognosis in ICU patients (16, 18–23), although some studies have small sample sizes. Therefore, with the help of the statistical power of meta-analysis, we aimed to conduct a systematic review and meta-analysis to explore the predictive value of PA in this patient population.

## Materials and methods

We performed the present meta-analysis according to the Preferred Reporting Items for Systematic Reviews and Meta-Analyses (PRISMA) statement (24; Supplementary material 1).

### Search strategy

Two authors (W-HZ and YY) independently searched the following electronic database from inception through Jan 31, 2022, without language restriction: PubMed, EMBASE, Web of Science, and the Cochrane Center Register of Controlled Trials (CENTRAL). We used Medical Subject Headings, keywords, and Emtree terms in the primary search. Studies that evaluated the PA on the prognosis of critically ill patients were included, regardless of study design. We also hand-searched the references list of relevant articles to identify potential studies that fulfill the eligibility criteria. Details information on the search strategy is summarized in Supplementary material 2.

### Selection criteria and outcomes

We considered including studies if they evaluated the critical adult patients (≥ 18 years) on any prognostic outcomes (i.e., mortality rate, survival time) by bioimpedance PA. Studies that used methods other than PA were excluded. We excluded studies recruiting children, breastfeeding women, transplantation, pregnant, or studies without reporting any prognostic outcomes. Animal studies, case reports, experimental models, editorials, and reviews were excluded. In addition, articles published only in abstract form or meeting reports were also excluded. At least two authors (YY, W-HZ, and H-BH) examined and agreed with the studies’ final inclusion.

The primary outcome was all-cause mortality at the longest follow-up available. Secondary outcomes included duration of MV, length of stay (LOS) in ICU or hospital, and adverse events (AEs, defined by each study author).

### Data extraction and quality assessment

Two authors (YY and Y-HZ) independently extracted the following information from included studies: the study characteristics (first author, country, publish year, study design, and sample size); patient characteristics (age, male, disease severity, population, and body mass index), PA parameters, and predefined outcome. YY and Y-HZ also independently evaluated potential evidence of bias using the Newcastle-Ottawa quality assessment scale for cohort studies (25). A score ≤ 5, a score of 6 or 7, and a score ≥ 8 were considered low, medium, and high quality, respectively. Discrepancies were identified and resolved by consensus or discussion with a senior author (H-BH).

### Data analysis

The results were combined to estimate the pooled odds ratio (OR) and associated 95% confidence intervals (CI) for dichotomous outcomes. As to the continuous outcomes, mean differences (MD) and 95% CI were estimated. We calculated pooled estimates and proportions with 95% CI using the Freeman-Tukey double-arcsine transformation. Some studies reported the median as the measure of treatment effect, with an accompanying interquartile range (IQR). We estimated the mean from the median and standard deviations (SD) from IQR (26).

According to the different reporting forms of PA provided by the included studies, we separately conducted three types of meta-analyses for the risk estimation between PA and all-cause mortality in critically ill patients: (1) We compared the baseline PA values between survival and non-survival groups. (2) We compared the all-cause mortality rate between the low and normal PA groups. (3) As to studies utilizing regression analyses to investigate the relationship between baseline PA (as a continuous or categorical variable) and mortality, we combine the mortality estimates with corresponding standard errors by the generic inverse variance method. Thus, these studies’ OR and hazard ratio (HR) required natural logarithmic transformations before merging. When both multivariate and univariate results were available, the former was preferred in the present analysis.

We tested between-study statistical heterogeneity using the *I*^2^ statistic. An *I*^2^ < 50% indicates insignificant heterogeneity, and a fixed-effect model was used, whereas a random-effect model was used in cases of significant heterogeneity (*I*^2^ > 50%). Publication bias was assessed by visual inspection of funnel plots. All statistical analyses were performed with Review Manager Version 5.3, and significance testing was at the two-tailed 0.05 level.

### Additional analyses

To explore the potential influence factors for the primary outcome, we performed subgroup analyses by pooling studies with the following properties: (1) Geographic location: Asian, America, or Europe; (2) Sample size: > 200 or ≤ 200; (3) Study design: Prospective or retrospective; (4) Selected ICU patients or not, and (5) Mortality prevalence: mortality rate < 20%, or > 20%. Additionally, we conducted sensitivity analyses by excluding one study at a time to explore whether an individual study’s particular result drove the results.

## Results

### Trial identification and characteristics

**FIGURE 1:**
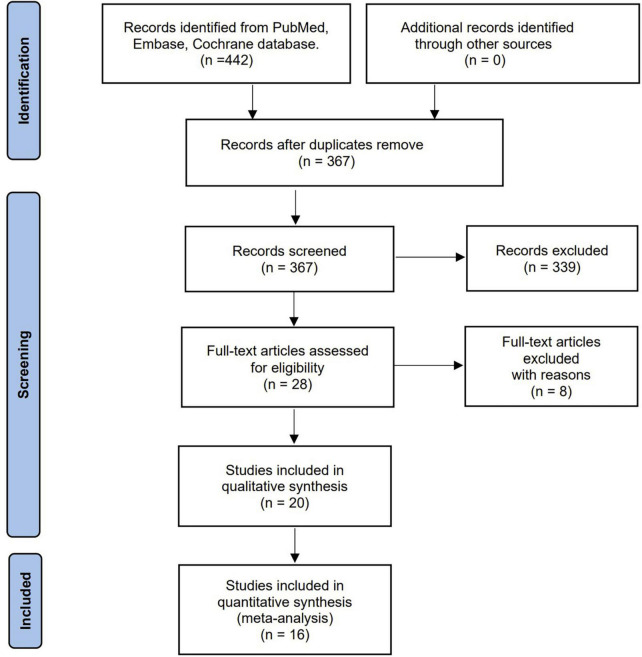
Flow diagram for the identification of relevant studies.

Our literature search yielded 442 potentially eligible articles through database searching. Further screening of 28 full texts identified 20 studies with 3,770 patients that fulfilled our inclusion criteria and were included in the final analysis (13–23, 27–35). Figure 1 shows the search strategy flowchart. We excluded eight studies summarized in Supplementary material 2 with exclusion reasons based on the full-text evaluation while presenting the main characteristics of the included studies in Table 1. These studies were published from 2012 to 2022. Sixteen out of the 20 studies were single-center studies. All the included studies recruited adult patients with sample sizes ranging from 31 to 931 cases. We extracted the PA measurements (i.e., PA of the total cohort, male, female, survival, and non-survival groups) and cut-off definitions used from the included studies (Tables 1, 2). All the included studies but one (16) provided the exact timing of PA measurement, while three (14, 18, 22) reported repeated measures after ICU admission (Supplementary material 3). The bioelectrical impedance analysis/phase angle methods among the included studies were summarized in the Supplementary material 3. In addition, six included studies (13, 18, 20, 21, 29, 32) reported the correlation between disease severity (SOFA, APACHE II, SAPS II, and SAPS III) and PA (Supplementary material 4). However, the pooled results were unavailable due to the few included studies. Of note, eight included studies (15–17, 19, 20, 29, 30, 33) evaluated the value of PA applications in nutrition, with different objectives [i.e., using PA in identifying malnutrition (15, 17), assessing nutritional status (16, 20, 29, 30), exploring PA as a predictor of nutrition risk (19, 33)], various nutritional assessment tools [i.e., subjective global assessment (15, 30), fat-free mass index (33), and NUTRIC score (19, 20, 29), SM-CSA (17), or serum albumin level and total lymphocyte count (16)], and different outcomes presented. Overall, most of these studies affirmed the value of PA in terms of nutrition for critically ill patient’s Supplementary material 5.

**TABLE 1:** Characteristics of included studies in the current meta-analysis.

Study	Country	Design	Population	Cut-off Rf	Sample size	Age, year	Male, %	Disease severity	Mortality^a^
Visser et al. (33)	Netherlands	P, SC	SICU	30th percentile	325	66	82	E > 6: 40%	Postoperative
Berbigier et al. (13)	Brazil	R, SC	Sepsis	NA	50	65	58	A 23; S 8	ICU
da Silva et al. (28)	Brazil	R, SC	Mixed ICU	ROC curve	95	64	63	A 17; S 6	ICU
Lee et al. (16)	Korea	R, SC	Mixed ICU	NA	66	63	64	A 16	Hospital
Vermeulen et al. (21)	Brazil	CS, SC	Mixed ICU	Previous study	35	56	74	A 10; S 3	Hospital
Thibault et al. (18)	France	P, MC	Mixed ICU	NA	931	61	60	A 19	28-days
Kuchnia et al. (17)	USA	P, MC	Mixed ICU	15th percentile	71	57	62	A 16; S 5	ICU, hospital
Stapel et al. (34)	Netherlands	P, SC	Mixed ICU	ROC curve	196	65	67	A 23; S 8	90-days
Lee et al. (14)	Korea	P, SC	SICU	None	241	63	67	A 16; S 7	Hospital
Buter et al. (29)	Netherlands	P, SC	Mixed ICU	None	299	66	66	A 14	Hospital
Ellegard et al. (22)	Sweden	R, SC	Mixed ICU	None	52	66	67	S 8	ICU
do Amaral Paes et al. (20)	Brazil	P, SC	Critical CA	ROC curve	31	61	48	A 15; S 3	1-year
Razzera et al. (19)	Brazil	P, SC	Critical CA	ROC curve	87	63	49	A 24; S 7	Hospital
Jansen et al. (15)	Brazil	P, MC	Mixed ICU	Previous study	169	60	57	A 19	Hospital
Yao et al. (23)	China	R, SC	Mixed ICU	ROC curve	201	49	61	A 15; S 8	90-days
Yasui-Yamada et al. (30)	Japan	R, SC	Critical CA	25th percentile	501	70	63	NA	5-years
Osuna-Padilla et al. (32)	Mexico	P, SC	COVID	ROC curve	67	55	76	A 21; S 9	60-days
Ko et al. (27)	Korea	P, SC	MICU	Previous study	97	62	58	A 19; S 8	Hospital
da Silva Passos et al. (31)	Brazil	P, SC	SICU	ROC curve	160	43	76	A 17; S 9	28-days
Formenti et al. (35)	Italy	P, SC	Mixed ICU	NA	96	69	68	A 26; S 7	ICU

^a^Defined as mortality rate of longest follow-up. A, acute physiology and chronic health evaluation; CA, cancer; CS, cross-section; E, euro score; ICU, intensive care unit; MC, multiple-centers; NOS, Newcastle-Ottawa scale; MICU, medical ICU; NA, not available; P, prospective; PA, phase angle; R, retrospective; S, Sequential Organ Failure Assessment; SC, single-center.

**TABLE 2:** Phase angle (PA) levels in the included studies on admission.

Study	Average PA, ^°^	PA in male, ^°^	PA in female, ^°^	PA in survivors, ^°^	PA in non-survivors, ^°^
Visser et al. (33)	5.9 ± 1.0				
Berbigier et al. (13)	5.4 ± 2.6	5.4 ± 1.9	4.1 ± 1.3		
da Silva et al. (28)	4.9 ± 1.4	5.30 ± 1.33	4.24 ± 1.2	< 5.1, (58%) > 5.1, (42%)	< 5.1, (67%) > 5.1, (33%)
Lee et al. (16)	4.0 ± 1.2			4.1 ± 1.2	2.9 ± 0.8
Vermeulen et al. (21)	4.2 ± 1.0			< 5.1, (69%) > 5.1, (31%)	< 5.1, (100%)
Thibault et al. (18)	4.5 ± 1.9			4.59 ± 1.79	4.10 ± 2.04
Kuchnia et al. (17)	4.3 ± 1.4	4.54 ± 1.36	4.01 ± 1.42		
Stapel et al. (34)	4.9 ± 1.3			5.0 ± 1.3	4.1 ± 1.2
Lee et al. (14)	4.0 ± 1.4			4.1 ± 1.3	3.2 ± 1.5
Buter et al. (29)	4.6 ± 1.2	5.5 ± 1.2	5.0 ± 1.4		
Ellegard et al. (22)	3.7 ± 1.0				
do Amaral Paes et al. (20)	4.0 ± 1.5	4.6 (3.5–5.5)	3.7 (3.1–4.5)	4.7 (3.8–5.5)	3 (2.4–3.7)
Razzera et al. (19)	5.4 ± 1.7			5.6 ± 1.1	5.2 ± 2.2
Jansen et al. (15)	5.3 ± 1.7	5.75 ± 1.83	4.82 ± 1.40		
Yao et al. (23)	3.6 (2.7–4.8)			4.1 (3.1–5.3)	3.1 (2.4–3.8)
Yasui-Yamada et al. (30)	4.7 (4.2–5.3)	5.0 (4.4–5.5)	4.4 (4.0–4.8)		
Osuna-Padilla et al. (32)	5.0 ± 1.2			5.4 ± 1.2	4.4 ± 1.0
Ko et al. (27)	3.6 ± 1.2			4.9 ± 1.2	4.4 ± 1.5
Passos et al. (31)	4.9 ± 1.2				
Formenti et al. (35)	3.8 ± 2.2				

Data were expressed as mean ± SD or median (IQR). PA, phase angle.

The quality of the included studies was moderate to high, and the Newcastle-Ottawa Score for the quality of the included studies was summarized in Supplementary material 6.

### Data analyses

**FIGURE 2:**
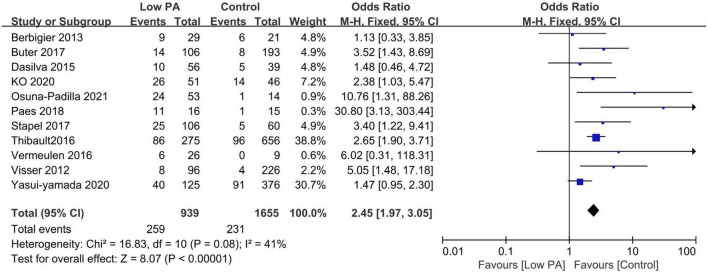
Forest plot showing the mortality rate in the lower and normal phase angle groups in critically ill patients and the pooled estimates.

All included studies provided survival information. Eleven studies with 2,594 patients reported all-cause mortality between low and normal PA groups (13, 18, 20, 21, 27, 29–34). Among these patients, 939 had low PA, and 259 died (27.6%) compared to 231 deaths (14.0%) in 1,655 normal PA patients. Low PA was associated with a significantly higher risk of mortality (OR 2.45, 95% CI 1.97–3.05, *P* < 0.00001), with heterogeneity of 41% observed (Figure 2). Subsequently, we conducted subgroup analyses to explore potential heterogeneity sources. In terms of between-groups mortality analyses, low PA was associated with higher mortality risk in all the predefined subgroups except the long-term follow-up group with only two studies (Table 3).

**TABLE 3:** Subgroup analysis on association between phase angle (PA) and mortality in critically ill patients.

Subgroup analysis		References	Patient number	Odds ratio (95% CI)	*I* ^2^	*p*
**Mortality (low vs. normal PA groups)**						
Sample size	> 200	(18, 29, 30, 33)	2,053	2.45 [1.55, 3.89]	57%	0.00001
	< 200	(13, 20, 21, 27, 28, 32, 34)	541	2.93 [1.85, 4.64]	38%	<0.00001
Geographic location	Asian	(27, 30)	598	1.64 [1.11, 2.43]	0%	0.01
	Not Asian	(13, 18, 20, 21, 28, 29, 32–34)	1,996	2.94 [2.26, 3.84]	26%	<0.00001
Design	Prospective	(18, 20, 21, 27, 29, 32, 34)	1,626	3.07 [2.34, 4.03]	10%	<0.00001
	Retrospective	(13, 28, 30, 33)	968	1.62 [1.12, 2.34]	22%	0.01
Follow-up period	Long-term	(20, 30)	532	5.45 [0.28, 106]	87%	0.26
	Short-term	(13, 18, 21, 27–29, 32–34)	2,062	2.75 [2.13, 3.56]	0%	<0.00001
Unselected ICU	Not	(13, 20, 27, 30, 32, 33)	1,068	2.89 [1.39, 6.01]	62%	0.004
Patients	Yes	(18, 21, 28, 29, 34)	1,526	2.72 [2.04, 3.64]	0%	<0.00001
Mortality prevalence	< 20%	(18, 21, 28, 29, 33, 34)	1,848	2.82 [2.13, 3.73]	0%	<0.00001
	> 20%	(13, 20, 27, 30, 32)	764	2.60 [1.16, 5.81]	62%	0.02
**PA values (survivors vs. non-survivors)**						
Sample size	> 200	(14, 18, 23)	1,373	0.78 [0.44, 1.11]	57%	<0.00001
	< 200	(16, 19, 20, 31, 32, 34)	607	0.91 [0.66, 1.15]	40%	<0.00001
Geographic location	Asian	(14, 16, 23)	508	1.00 [0.74, 1.26]	0%	<0.00001
	Not Asian	(18–20, 31, 32, 34)	1,472	0.77 [0.46, 1.09]	52%	<0.00001
Design	Prospective	(18–20, 31, 32, 34)	508	1.00 [0.74, 1.26]	0%	<0.00001
	Retrospective	(14, 16, 23)	1,472	0.77 [0.46, 1.09]	52%	<0.00001
Follow-up period	Long-term	(20)	31	1.70 [0.88, 2.52]	-	<0.0001
	Short-term	(14, 16, 18, 19, 23, 31, 32, 34)	1,949	0.78 [0.62, 0.95]	24%	<0.00001
Unselected ICU	Not	(16, 18, 23, 34)	1,394	0.85 [0.53, 1.16]	53%	<0.00001
Patients	Yes	(14, 19, 20, 31, 32)	586	0.86 [0.60, 1.12]	46%	<0.00001
Mortality prevalence	< 20%	(14, 16, 18, 34)	1,434	0.75 [0.53, 0.97]	42%	<0.00001
	> 20%	(19, 20, 23, 31, 32)	546	0.91 [0.66, 1.15]	48%	<0.00001

HR, hazard ratio; ICU, intensive care unit; LOS, length of stay; OR, odds ratio; PA, phase angle.

**FIGURE 3:**
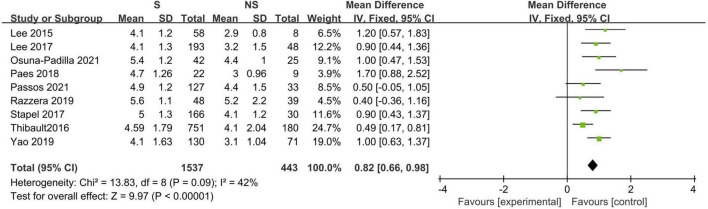
Forest plot showing the standardized mean phase angle values in the death and survival groups and the pooled estimates.

Eleven studies described the baseline PA between survivors and non-survivors, and nine of these studies provided available pooled data (14, 16, 18–20, 23, 31, 32, 34). When pooling, non-surviving patients had lower PA values than surviving patients during the follow-up period (*N* = 1,980; MD 0.82°, 95% CI 0.66–0.98, *I*^2^ = 42%; *P* < 0.00001, Figure 3). The subgroup analyses results are presented in Table 3, and a significant association was consistent in all the defined subgroups.

**FIGURE 4:**
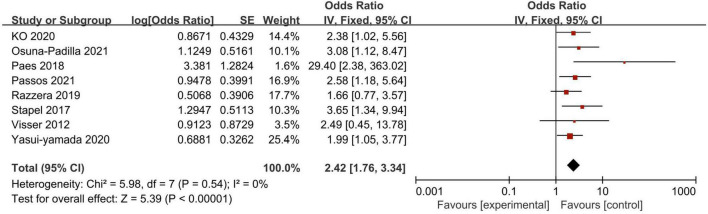
Forest plot demonstrating the association between phase angle (as categorical variable) and mortality in critically ill patients and the pooled estimates.

**FIGURE 5:**
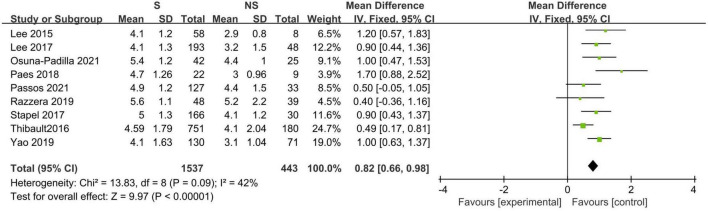
Forest plot demonstrating the association between phase angle (as continuous variable) and mortality in critically ill patients and the pooled estimates.

A total of 11 studies investigated the association between PA and mortality of ICU patients using logistic regression analysis (as a continuous or categorical variable). The pooled data showed that PA (as a continuous variable) had a significant prognostic role on patients’ survival (7 studies, *N* = 2,234; OR = 0.64; 95% CI 0.52–0.79; *P* < 0.00001, *I*^2^ = 73%, random-effects model, Figure 4; 14, 18, 23, 27, 30, 32, 34). Similarly, belonging to the reduced PA group (as a categorical variable) was a significant risk factor for mortality (8 studies, *N* = 1,464; OR = 2.42; 95% CI 1.76–3.34; *P* < 0.00001, *I*^2^ = 0%, fixed-effects model, Figure 5; 19, 20, 27, 30–34). Table 4 shows the detailed information of subgroup analyses by categories or continuous variables, and the significant association between PA and all-cause mortality was also confirmed in all subgroups except the long-term follow-up group with only two studies.

**TABLE 4:** Subgroup analysis on the association between phase angle (PA) and mortality in critically ill patients.

Subgroup analysis		References	Patient number	Odds ratio (95% CI)	*I* ^2^	*p*
**Regression analyses (PA as a continuous variable)**						
Sample size	> 200	(14, 18, 23, 30)	1,874	0.69 [0.56, 0.86]	78%	0.001
	< 200	(27, 32, 34)	360	0.53 [0.39, 0.73]	27%	<0.00001
Geographic location	Asian	(14, 23, 27, 30)	1,040	0.61 [0.48, 0.78]	56%	0.0001
	Not Asian	(18, 32, 34)	1,194	0.62 [0.42, 0.98]	77%	0.04
Design	Prospective	(14, 18, 27, 32, 34)	1,532	0.59 [0.42, 0.83]	77%	0.002
	Retrospective	(23, 30)	702	0.67 [0.49, 0.92]	71%	0.01
Follow-up period	Long-term	(30)	501	0.56 [0.42, 0.75]	-	<0.0001
	Short-term	(14, 18, 23, 27, 32, 34)	1,733	0.66 [0.53, 0.82]	71%	0.0002
Unselected ICU	Not	(14, 27, 30, 32)	1,040	0.52 [0.42, 0.63]	0%	<0.00001
Patients	Yes	(18, 23, 34)	1,194	0.83 [0.76, 0.91]	77%	<0.0001
Mortality prevalence	< 20%	(14, 18, 34)	1,368	0.69 [0.50, 0.96]	77%	0.03
	> 20%	(23, 27, 30, 32)	866	0.57 [0.41, 0.80]	65%	0.001
**Regression analyses (PA as a categorical variable)**						
Sample size	> 200	(30, 33)	826	2.05 [1.12, 3.72]	0%	0.02
	< 200	(19, 20, 27, 31, 32, 34)	638	2.59 [1.77, 3.80]	9%	<0.00001
Geographic location	Asian	(27, 30)	598	2.12 [1.27, 3.54]	0%	0.004
	Not Asian	(19, 20, 31–34)	866	2.64 [1.75, 4.00]	8%	<0.00001
Design	Prospective	(19, 20, 27, 31–34)	963	2.59 [1.78, 3.76]	0%	<0.00001
	Retrospective	(30)	501	1.99 [1.05, 3.77]	-	0.03
Follow-up period	Long-term	(20, 30)	532	5.75 [0.44, 75.8]	75%	0.18
	Short-term	(19, 27, 31–34)	932	2.45 [1.68, 3.57]	0%	<0.00001
Unselected ICU	Not	(19, 20, 27, 30–33)	1,268	2.31 [1.64, 3.25]	0%	<0.00001
Patients	Yes	(34)	196	3.65 [1.34, 9.94]	-	0.01
Mortality prevalence	< 20%	(33, 34)	521	3.31 [1.39, 7.86]	0%	<0.00001
	> 20%	(19, 20, 27, 30–32)	943	2.30 [1.63, 3.26]	0%	<0.00001

ICU, intensive care unit; LOS, length of stay; OR, odds ratio; PA, phase angle.

Further sensitivity analyses by excluding one study at a time showed no change in the previous results (data not shown). In addition, we found no evidence of publication bias with the funnel plots that did not suggest asymmetry (Supplementary material 7).

## Discussion

The current study is the first systematic review and meta-analysis to investigate the predictive value of BIA-derived PA in the prognosis of critically ill patients. Our results showed the baseline PA varied in patients after ICU admission, ranging from approximately 3.7° to 5.9°. PA was an independent risk factor for all-cause mortality in the ICU setting with a nearly 1.5-fold increase. Further subgroup analyses and sensitivity analyses confirmed this finding. In addition, reduced PA is related to disease severity, more extended hospital LOS and longer duration of MV.

Our study has several advantages. The current meta-analysis provides strong evidence that fills a gap in previous guidelines (36). That is, clinicians can use PA to predict the prognosis in the ICU setting. Second, our findings are consistent with earlier findings in other patient populations, including advanced tumors, cirrhosis, renal failure, transplantation, and surgical patients (9–12). Therefore, our meta-analysis adds a new population of evidence. Third, most included studies focused on non-selected critically ill patients in the ICU (15–18, 21, 22, 28, 29, 34), making our findings more generalizable. Fourth, we thoroughly assessed mortality risk, including mortality between low PA and control and a linear relationship between PA and mortality. In addition, we included 20 studies of more than 3,700 patients with sufficient statistical power to perform subgroup analyses and sensitivity analyses based on different potential influencing factors. The results were consistent, further supporting the robustness of our main results.

Our results showed that ICU patients had lower PA measurements than healthy individuals, with the mean PA varied among the included studies (from approximately 3.7° to 5.9°) (13–22, 28–34). The variation was related to different patient characteristics, such as ethnicity, gender ratio, disease type, age, etc. For example, we found that the mean PA in the Asian population was 3.6° (3.0°–4.7°) (14, 16, 23, 30), lower than that of European and American patients of 5.1° (4.6°–5.9°) (13, 15, 17–22, 27–29, 31–34). Similar to the data in the healthy population, PA was significantly higher in male patients in the ICU, which was related to the higher muscle reserve in males than in females (Table 2). In addition, PA might be affected by some treatment or internal environmental changes in the body during the ICU stay (7, 8). Commonly seen was a substantial fluid transfer before ICU admission or within the first few hours of ICU admission may lead to changes in PA, which reflects inflammation-induced changes in membrane integrity and causes fluid to redistribute into the extracellular space (37). The effect of altering hydration on PA may explain why in Thibault et al.’s study (18), PA on day first but not on day five after admission could predict mortality. Thus, early PA measurement after admission may reduce the confounding of hydration changes. Finally, it should be noted that some included retrospective studies only included patients with PA measured, so that their reports may underestimate the PA incidences (13, 16, 22, 23, 28, 30, 33).

Phase angle (PA) reflects cell membrane integrity, permeability, and fat-free mass (38). Thus, lower PA can indicate severely compromised cell membrane integrity and increase cell membrane permeability due to acute disease (i.e., membrane dysfunction and fluid transfer) and the effects of underlying systemic conditions. Poorer cellular health, cellular dysfunction, and nutritional status worsen. As shown in some included studies (15–17, 19, 20, 29, 30, 33), PA is used in the assessment of nutritional status in critically ill patients and has been shown to be an accurate indicator of nutritional risk screening. In addition, PA measurement declines with age and sarcopenia, and low PA is associated with malnutrition and frailty (39, 40). In this respect, PA may reflect limited physiological reserves, which explains its association with long-term mortality (34). Therefore, reduced PA reflects acute changes and underlying poor health, muscle wasting, and fragility, which are poorly captured by other disease severity predictors commonly used in the ICU, such as the APACHE II score (14, 34).

However, our results require further discussion. First, we found a lack of a unified definition of PA cut-offs among the included studies, leading to an important source of heterogeneity in our results. Most authors adopted PA cut-off points reported in previous research to define reduced PA (15, 21, 27) or based on specific cut-off points [e.g., adopted the lowest quartile, the first quartile, or the median according to their cohorts (17, 30, 33)]. Although these studies were conducted in different cohorts, they provided consistent results, associating low PA values with lower survival rates. Therefore, these results suggest that a reduced PA cut-off point is reasonable for predicting critical illness outcomes. However, defining a unified PA cut-off point is not easy since each study contains different diseases, and data cannot be fully extrapolated to different study cohorts. On the other hand, there are differences between studies in the equipment used, the electrodes used, and the frequency of measurements (Supplementary material 3), which affect the determination of standardization threshold. Given the differences in disease, ethnicity, body size, and diet among ICU patients, the validity and accuracy of cut-offs across geographic, ethnic, and disease states still need further confirmation.

Second, most included studies assessed baseline PA at ICU admission rather than described overall PA exposure to characterize the effect of PA changes on ICU patients. Only two studies added to this gap (18, 22). In a retrospective ICU cohort study, Ellegard and colleagues found that among 26 patients who were reassessed for PA 5 days after admission, PA increased by 0.62° ± 1.24 in 17 survivors and decreased by 0.24° ± 0.82° in 9 non-survivors, which resulted in a between-group difference of 0.86° (*P* = 0.048) (22). The authors concluded that repeated PA measurement in ICU patients could help predict clinical outcomes. Thibault et al. also reassessed the PA in their study (18). In 540 patients with PA measurements on day 5, approximately 0.3° was higher in survivors than non-survivors (18). Therefore, assessing changes in PA over the clinical course of ICU patients may be a more effective predictor of clinical outcome assessment than a single clinical outcome. On the other hand, the previous finding of a higher mortality rate in patients without PA improvement after treatment suggests that residual PA reduction still has a predictive value (18). Thus, the predictive value of PA may be related to its treatment responsiveness, which could help assess the long-term risk of death and could be used to monitor targeted interventions aimed at improving the long-term prognosis of ICU patients.

Third, two included studies focused on the septic population and came to different conclusions (21, 28). Silva and colleagues found that PA was a good prognostic marker for patients without sepsis but not for the septic cohort (28). Meanwhile, they observed a significant negative correlation between PA and APACHE II scores only in patients without sepsis (*r* = −0.506; *P* < 0.001). In contrast, the results of Vermeulen et al. suggest that PA showed no differences in patients between patients (*P* = 0.179) with or without sepsis and was a useful prognostic indicator in both groups of patients (21). Of note, two studies had small sample sizes (< 50) and used sepsis-2 and sepsis-3 diagnostic criteria. Moreover, compared to Silva et al. (28), Vermeulen et al. included sicker patients (APACHE II: 22 vs. 10), less male ratio (26 vs. 63%), more from surgical settings (60 vs. 36%), and younger (55 vs. 65%) in their cohort (21). All of these may all contribute to the differences between the two studies.

Additionally, most included studies have focused on the differences in PA between men and women and among patients over 60 years of age or older, while few PA cut-off points based on gender and age have been suggested. Currently, standardized PA (SPA) normalized for age, gender, and BMI has been proposed, with the calculation = (measured PA − mean population reference PA)/standard deviation of the reference population. However, only two included studies described the associations between PA indicators obtained by BIA with mortality (15, 32). Jensen et al. reported that reduced SPA increased about three times the chance of having malnutrition (OR = 2.79, 95% CI 1.39–5.61) and two times the chance of prolonged hospital stay (OR = 2.27; 95% CI 1.18–4.34) (15). In the other study by Osuna-Padilla et al. (32), the authors found that SPA and PA were significant predictors of 60-day mortality (OR, 0.45; *P* = 0.001). SPA may be a better prognostic predictor and should deserve more clinical attention.

Our meta-analysis has several limitations. (1) The observational design of all included studies excluded any causal inference. Meanwhile, only patients who underwent PA measurements were recruited, prone to selection bias. (2) Most studies assessed only baseline PA levels at ICU admission, ignoring assessments of PA levels over time. (3) We included several small studies and most were single-center designs. (4) Most included studies focused on unselected critically ill patients, and the uneven distribution of different underlying diseases in these studies may also exert different prognostic values. (5) In subgroup and sensitivity analyses, we could not have considered all confounding factors that may play a role in the relationship between PA and ICU mortality, such as the timing of measurement, age, nutritional status, and the effect of artificial feeding. (6) Only a few studies have proposed the severity of PA abnormalities and their impact on prognosis, but the further investigation could not be done due to a lack of grading criteria. (7) Although we included 20 studies in our manuscript, the clinical application of PA was limited to research due to the lack of knowledge and available instrumentation. Moreover, the different instruments, frequencies, and electrodes used might potentially bias the mean values among the included studies finally, it remains unclear whether PA-guided therapy reduces mortality in ICU patients. Thus, further studies on this topic are needed.

## Conclusion

The findings of the current meta-analysis suggest that PA may be an important prognostic factor of survival in this population and nicely complement the deficiencies of other severity scoring systems. However, it should be noted that the included studies used different cut-off values, which was the primary source of the existing heterogeneity. Therefore, further studies are needed to define the optimal cut-off value to define PA according to geography, race, and disease and to further confirm our findings.

## Data availability statement

The original contributions presented in this study are included in the article/Supplementary material, further inquiries can be directed to the corresponding author.

## Author contributions

W-HZ contributed to the search of the scientific literature and drafted the manuscript. Y-HZ contributed to the conception, design, and data interpretation. YY helped to collect the data and performed statistical analyses. H-BH contributed to the conception, design, data interpretation, manuscript revision for critical intellectual content, and supervision of the study. All authors contributed to the article and approved the submitted version.
