# Supplementary material for: Prognostic role of bioelectrical impedance phase angle for critically ill patients: A systemic review and meta-analysis
Source: Front Med (Lausanne). 2023 Jan 9;9:1059747. doi: 10.3389/fmed.2022.1059747 (PMC9868673; doi:10.3389/fmed.2022.1059747)
Supplement: Supplementary file 1 [file Data_Sheet_1.docx]

**Prognostic role of phase angle for critically ill patients: a systemic review and meta-analysis**

**Supplementary material**

1. Supplementary material 1

Checklist following PRISMA guideline………………………………………………………………………………………………………..1

2. Supplementary material 2

Search strategy………………………………………………………………………………………………….….……………………..…..………..5

Table S2: Studies needed for full-reviewed but not included in the current meta-analysis (n=8 trials)………7

3. Supplementary material 3

The bioelectrical impedance analysis/phase angle methods among the included studies…………………...…….8

4. Supplementary material 4

Table S4-1: Comparison of disease severity in patients with a high and low phase angle….……..………………11

Table S4-2: Correlation between the Phase Angle (°) and the disease severity………………………..………………..11

5 Supplementary material 5

Evaluation of the value of PA applications in nutrition among the included studies.….…………………….………13

6. Supplementary material 6

Table S5: Quality assessment and overall risk of bias of included studies………………….…………………………......14

7. Supplementary material 7

Figure S6: Publication bias………………………………………..………………………………………………………….….….……………15

**Supplementary material 1**

**PRISMA 2009 checklist**

| **Section/topic** | **#** | **Checklist item** | **Reported on page #** |
| --- | --- | --- | --- |
| **TITLE** | | |  |
| Title | 1 | Identify the report as a systematic review, meta-analysis, or both. | 1 |
| **ABSTRACT** | | |  |
| Structured summary | 2 | Provide a structured summary including, as applicable: background; objectives; data sources; study eligibility criteria, participants, and interventions; study appraisal and synthesis methods; results; limitations; conclusions and implications of key findings; systematic review registration number. | 2 |
| **INTRODUCTION** | | |  |
| Rationale | 3 | Describe the rationale for the review in the context of what is already known. | 4 |
| Objectives | 4 | Provide an explicit statement of questions being addressed with reference to participants, interventions, comparisons, outcomes, and study design (PICOS). | 5 |
| **METHODS** | | |  |
| Protocol and registration | 5 | Indicate if a review protocol exists, if and where it can be accessed (e.g., Web address), and, if available, provide registration information including registration number. | 6 |
| Eligibility criteria | 6 | Specify study characteristics (e.g., PICOS, length of follow-up) and report characteristics (e.g., years considered, language, publication status) used as criteria for eligibility, giving rationale. | 6 |
| Information sources | 7 | Describe all information sources (e.g., databases with dates of coverage, contact with study authors to identify additional studies) in the search and date last searched. | 6 |
| Search | 8 | Present full electronic search strategy for at least one database, including any limits used, such that it could be repeated. | 6 and Appendix file 2 |
| Study selection | 9 | State the process for selecting studies (i.e., screening, eligibility, included in systematic review, and, if applicable, included in the meta-analysis). | 6-7 |
| Data collection process | 10 | Describe method of data extraction from reports (e.g., piloted forms, independently, in duplicate) and any processes for obtaining and confirming data from investigators. | 7 |
| Data items | 11 | List and define all variables for which data were sought (e.g., PICOS, funding sources) and any assumptions and simplifications made. | 7 |
| Risk of bias in individual studies | 12 | Describe methods used for assessing risk of bias of individual studies (including specification of whether this was done at the study or outcome level), and how this information is to be used in any data synthesis. | 7 |
| Summary measures | 13 | State the principal summary measures (e.g., risk ratio, difference in means). | 7 |
| Synthesis of results | 14 | Describe the methods of handling data and combining results of studies, if done, including measures of consistency (e.g., I^2^) for each meta-analysis. | 7-8 |

| Risk of bias across studies | 15 | Specify any assessment of risk of bias that may affect the cumulative evidence (e.g., publication bias, selective reporting within studies). | 7 |
| --- | --- | --- | --- |
| Additional analyses | 16 | Describe methods of additional analyses (e.g., sensitivity or subgroup analyses, meta-regression), if done, indicating which were pre-specified. | 8 |
| **RESULTS** | | |  |
| Study selection | 17 | Give numbers of studies screened, assessed for eligibility, and included in the review, with reasons for exclusions at each stage, ideally with a flow diagram. | 9  Figure 1 |
| Study characteristics | 18 | For each study, present characteristics for which data were extracted (e.g., study size, PICOS, follow-up period) and provide the citations. | 9  Table 1 |
| Risk of bias within studies | 19 | Present data on risk of bias of each study and, if available, any outcome level assessment (see item 12). | 9 |
| Results of individual studies | 20 | For all outcomes considered (benefits or harms), present, for each study: (a) simple summary data for each intervention group (b) effect estimates and confidence intervals, ideally with a forest plot. | 9 |
| Synthesis of results | 21 | Present results of each meta-analysis done, including confidence intervals and measures of consistency. | 10 |
| Risk of bias across studies | 22 | Present results of any assessment of risk of bias across studies (see Item 15). | Appendix 4 |
| Additional analysis | 23 | Give results of additional analyses, if done (e.g., sensitivity or subgroup analyses, meta-regression [see Item 16]). | 10 |
| **DISCUSSION** | | |  |
| Summary of evidence | 24 | Summarize the main findings including the strength of evidence for each main outcome; consider their relevance to key groups (e.g., healthcare providers, users, and policy makers). | 11 |
| Limitations | 25 | Discuss limitations at study and outcome level (e.g., risk of bias), and at review-level (e.g., incomplete retrieval of identified research, reporting bias). | 14 |
| Conclusions | 26 | Provide a general interpretation of the results in the context of other evidence, and implications for future research. | 15 |
| **FUNDING** | | |  |
| Funding | 27 | Describe sources of funding for the systematic review and other support (e.g., supply of data); role of funders for the systematic review. | 20 |

**Supplementary material 2**

**Search Strategy : (Database: PubMed Embase Cochrane library ; Search completed 20th Jan 2022)**

--------------------------------------------------------------------------------------------------------------------------------------------------------------------------------------------------------

**PubMed**

#1.

((((((((((((((((Electric Impedance) OR Impedance, Electric) OR Electrical Impedance) OR Impedance, Electrical) OR Impedance) OR Electric Resistance) OR Resistance, Electric) OR Electrical Resistance) OR Resistance, Electrical) OR Ohmic Resistance) OR Ohmic Resistances) OR Resistance, Ohmic) OR Resistances, Ohmic) OR Bioelectrical Impedance) OR Impedance, Bioelectrical) OR Biolectric Impedance) OR Impedance, Biolectric

#2

phase angle

#3.

(("Critical Care"[Mesh]) OR ((((critical care[Title/Abstract]) OR (critically ill[Title/Abstract])) OR (intensive care[Title/Abstract])) OR (((((((((((((((Critical Illness[Title/Abstract]) OR (Critical Care[Title/Abstract])) OR (intensive care units[Title/Abstract])) OR (Burn units[Title/Abstract])) OR (coronary care units[Title/Abstract])) OR (respiration, artificial[Title/Abstract])) ) OR (ventilators, mechanical[Title/Abstract])) OR (pulmonary ventilation[Title/Abstract])) OR (respiratory insufficiency[Title/Abstract])) OR (multiple organ failure[Title/Abstract])) OR (systemic inflammatory response syndrome[Title/Abstract])) OR (respiratory distress syndrome, adult[Title/Abstract])) OR (sepsis[Title/Abstract])) OR (shock, septic[Title/Abstract]))))

#4. #1 AND #2 AND #3

**Embase**

#1 'septic shock' OR 'multiple organ failure*' OR 'multiple organ dysfunction*' OR 'systemic inflammatory response' OR 'respiratory distress syndrome*' OR 'respiratory care unit*' OR 'coronary care unit*' OR 'burn unit*' OR 'high dependency unit*' OR 'intensive therapy unit' OR 'intensive treatment unit*' OR 'intensive care' OR 'critical* ill*' OR 'critical care' /mp

#2 'lung ventilation' OR 'systemic inflammatory response syndrome' OR 'adult respiratory distress syndrome' OR 'septic shock' OR 'coronary care unit' OR 'intensive care unit' OR 'critically ill patient' OR 'intensive care'/exp

#3 'impedance' or 'electric impedance' or 'electrical impedance' or 'input impedance'

#4 'phase angle'

#5 #1 AND #2 AND #3 AND #4

**Cochrane library**

ID Search

#1 ("intensive care"):ti,ab,kw (Word variations have been searched)

#2 ("critically ill"):ti,ab,kw (Word variations have been searched)

#3 ("critical care"):ti,ab,kw (Word variations have been searched)

#4 ("critical illness"):ti,ab,kw (Word variations have been searched)

#5 ("Burn"):ti,ab,kw (Word variations have been searched)

#6 ("acute respiratory distress syndrom"):ti,ab,kw (Word variations have been searched)

#7 ("truma"):ti,ab,kw (Word variations have been searched)

#8 ("septic shock"):ti,ab,kw (Word variations have been searched)

#9 #1 OR #2 OR #3 OR #4 OR #5 OR #6 OR #7 OR #8

#10 ("phase angle"):ti,ab,kw(Word variations have been searched)

#11 ("Electric Impedance"):ti,ab,kw(Word variations have been searched)

#12 ("input impedance"):ti,ab,kw(Word variations have been searched)

#13 ("Electric Resistance"):ti,ab,kw(Word variations have been searched)

#14 ("Ohmic Resistance"):ti,ab,kw(Word variations have been searched)

#15 ("Bioelectrical Impedance"):ti,ab,kw(Word variations have been searched)

#16 #11 OR #12 OR #13 OR #14 OR #15

#19 #9 AND #10 AND #16

**Supplementary material 2**

**Table S2:** **Studies needed for full-reviewed but not included in the current meta-analysis (n=8 trials)**

| No | Study | Reason of exclusion |
| --- | --- | --- |
| 1 | Baldwin CE, Fetterplace K, Beach L, Kayambu G, Paratz J, Earthman C, Parry SM. Early Detection of Muscle Weakness and Functional Limitations in the Critically Ill: A Retrospective Evaluation of Bioimpedance Spectroscopy. JPEN J Parenter Enteral Nutr. 2020 Jul;44(5):837-848. doi: 10.1002/jpen.1719. Epub 2019 Oct 3. PMID: 31583738.. | Irrelevant to the current research |
| 2 | Sunario J, Wibrow B, Jacques A, Ho KM, Anstey M. Associations Between Nutrition Markers and Muscle Mass on Bioimpedance Analysis in Patients Receiving Parenteral Nutrition. JPEN J Parenter Enteral Nutr. 2021 Jul;45(5):1089-1099. doi: 10.1002/jpen.1986. Epub 2020 Sep 12. PMID: 32740938. | Reported without predefined outcome |
| 3 | Kyle UG, Genton L, Pichard C. Low phase angle determined by bioelectrical impedance analysis is associated with malnutrition and nutritional risk at hospital admission. Clin Nutr. 2013 Apr;32(2):294-9. doi: 10.1016/j.clnu.2012.08.001. Epub 2012 Aug 14. PMID: 22921419. | Reported without ICU admission |
| 4 | Al-Kalaldeh M, Suleiman K, Al-Kalaldeh O. Prognostic Performance of NUTRIC Score in Quantifying Malnutrition Risk in the Critically Ill in Congruence with the Bioelectrical Impedance Analysis. Nutr Clin Pract. 2020 Jun;35(3):559-566. doi: 10.1002/ncp.10440. Epub 2019 Nov 11. PMID: 31713274. | Reported without predefined outcome |
| 5 | Denneman N, Hessels L, Broens B, Gjaltema J, Stapel SN, Stohlmann J, Nijsten MW, Oudemans-van Straaten HM. Fluid balance and phase angle as assessed by bioelectrical impedance analysis in critically ill patients: a multicenter prospective cohort study. Eur J Clin Nutr. 2020 Oct;74(10):1410-1419. doi: 10.1038/s41430-020-0622-7. Epub 2020 Apr 14. PMID: 32286534. | Reported without predefined outcome |
| 6 | Ribeiro HS, Coury NC, de Vasconcelos Generoso S, Lima AS, Correia MITD. Energy Balance and Nutrition Status: A Prospective Assessment of Patients Undergoing Liver Transplantation. Nutr Clin Pract. 2020 Feb;35(1):126-132. doi: 10.1002/ncp.10323. Epub 2019 Jun 13. PMID: 31190346. | Patients of liver transplant |
| 7 | Sunario J, Wibrow B, Jacques A, Ho KM, Anstey M. Associations Between Nutrition Markers and Muscle Mass on Bioimpedance Analysis in Patients Receiving Parenteral Nutrition. JPEN J Parenter Enteral Nutr. 2021 Jul;45(5):1089-1099. doi: 10.1002/jpen.1986. Epub 2020 Sep 12. PMID: 32740938. | Irrelevant to the current research |
| 8 | Bakshi N, Singh K. Nutrition assessment and its effect on various clinical variables among patients undergoing liver transplant. Hepatobiliary Surg Nutr. 2016 Aug;5(4):358-71. doi: 10.21037/hbsn.2016.03.09. PMID: 27500148; PMCID: PMC4960422. | Patients of liver transplant |

**Supplementary material 3**

**Table S3: The bioelectrical impedance analysis /phase angle methods among the included studies**

| Study | Measured timing | Equipment | Frequency  kHz | Current  Micro A | Electrodes used |
| --- | --- | --- | --- | --- | --- |
| Visser 2012^[33]^ | Within 48 hours of ICU admission | BodyScout | 5-1000 | NA | NA |
| Berbigier 2013^[13]^ | Within 48 hours of ICU admission | Biodynamics 450 | 50 | 800 | Electrodes were positioned in the dorsal surface of the right wrist, the third metacarpal bone, the anterior surface of the right ankle between the bone prominences, and the dorsal surface of the third metatarsal bone |
| Silva 2015^[28]^ | Within 48 hours of ICU admission | Biodynamics 450 | 50 | 800 | Electrode were placed on the dorsal surface of the right wrist, the third metacarpus, the anterior surface of the right ankle between the prominent portions of bones, and on the dorsal surface of the third metatarsus |
| Lee 2015^[16]^ | NA | InBody S10 | NA | NA | Placed on patient's thumbs and middle fingers and two sides of ankles |
| Vermeulen 2016^[21]^ | Within 24 hours of ICU admission | Quantum II | 50 | 800 | Electrodes were positioned in the middle of the dorsal surfaces of the hands and feet proximal to the metacarpal-phalangeal and metatarsal-phalangeal joints, respectively, and also medially between the distal prominences of the radius and the ulna and between the medial and lateral malleoli at the ankle |
| Thibault 2016^[18]^ | On day 1 and day 5 of ICU stay | Nutriguard M | 50 | 800 | Electrodes were placed on the dorsal side of the left hand, left wrist, left foot, and left ankle |
| Kuchnia 2016^[17]^ | Within 72 hours of the initial CT scan | QuadScan 4000 | NA | NA | Electrodes were placed on the hands and feet |
| Stapel 2017^[34]^ | Within 24 hours of ICU admission | BIA 101 Anniversary edition device | 50 | 400 | Two pairs of electrodes were placed (source and sensor electrodes), one pair on the dorsum of the hand and one pair on the dorsum of the ipsilateral foot |
| Lee 2017^[14]^ | Twice weekly (Monday and Thursday) | InBody S10 | 50 | NA | Eight adhesive electrodes were used: one on the most distal part of the third metacarpal bone of each hand, one on each wrist, one on the most distal part of the second metatarsal bone in each foot, and one on the central part of each ankle |
| Buter 2017^[29]^ | Within 24 hours of ICU admission | BIA 101 Anniversary Sport Edition analyzer | 50 | NA | Electrodes are placed on the wrist and dorsal site of the hand and on the ipsilateral ankle and forefoot |
| Ellegard 2018^[22]^ | At ICU admission and ICU discharge | Bioimpedance spectroscopy | 50 | NA | NA |
| Paes 2018^[20]^ | Within 24 hours of ICU admission | BIA-450 impedance analyzer | 50 | 800 | Four electrodes were then positioned on the skin after cleansing, between the prominences of the radius and the ulna; on the posterior surface of the right wrist and between the malleolus of the tibia and the fibula; and on the anterior surface of the right ankle |
| Razzera 2019^[19]^ | The first 72 hours of ICU admission | BIA 310 analyzer | NA | NA | Four disposable adhesive electrodes were placed on the dorsal surface of the right hand and foot on clean and dry skin |
| Jansen 2019^[15]^ | Within 48 hours of ICU admission | Biodynamics® (model 310E) | NA | NA | Four adhesive and disposable electrodes were placed on the dorsal surface of the hand and right foot on dry and disinfected skin at predetermined anatomical sites. |
| Yao 2019^[23]^ | Within 3 days after admission to the ICU | QuadScan 4000 | 50, 100, 200 | NA | Two electrodes were placed approximately 5 mm apart on the dorsal surface of the right wrist and ipsilateral ankle under a supine position with arms and legs in abduction to avoid contacts with the trunk |
| Yasui-Yamada 2020^[30]^ | During the period between admission and surgery | InBody 770 | 50 | NA | NA |
| Osuna-Padilla 2021^[32]^ | During the first 48 h of initiating MV | InBody S10 | NA | NA | Eight adhesive electrodes were used: one on each wrist, one on the distal part of the third metacarpal bone of each hand, one on the central part of each ankle, and one on the distal part of the second metatarsal bone in each foot |
| Ko 2020^[27]^ | Within 24 hours after enrollment | InBody S10 | 1、5、50、250、500, and1000 | NA | Electrodes were attached on each thumb, third finger, and ankle |
| Passos 2021^[31]^ | Within 48 hours after ICU admission | InBody S10 | 1, 5, 50, 250, 500, and 1000 | NA | Eight reusable contact electrodes, which were placed on the first and third fingers of both hands and the lateral and medial sides of both ankles, were used |
| Paolo 2022^[35]^ | At ICU admission | InBody S10 | 1, 5, 50, 250, 500, and 1000 | NA | Eight adhesive electrodes were used: one on each wrist, one on the distal part of the third metacarpal bone of each hand, one on the central part of each ankle, and one on the distal part of the second metatarsal bone in each foot |

ICU=intensive care unit; NA=not available; MV=mechanical ventilation

**Supplementary material 4**

**Table S4-1: Comparison of disease severity in patients with a high and low phase angle**

| Study | N | SOFA | | SAPS II | | SAPS III | | APACHE II | |
| --- | --- | --- | --- | --- | --- | --- | --- | --- | --- |
|  |  | PA-high | PA-low | PA-high | PA-low | PA-high | PA-low | PA-high | PA-low |
| Vermeulen 2016^[21]^ | 35 | 1.0 (0.0-2.0) | 4.0 (1.8-6.3) |  |  | 41 (33-59) | 31 (27-41) | 8.0 (6.0-9.5) | 13.5 (8.5-19.8) |
| Thibault 2016^[18]^ | 931 |  |  | 40.5 ± 18.3 | 48.0 ± 19.2 |  |  | 17.7 ± 8.7 | 21.8 ± 9.2 |
| Buter 2017^[29]^ | 299 |  |  |  |  |  |  | 13 [10-17] | 15 [12-19] |

APACHE II= Acute Physiology and Chronic Health Evaluation; SOFA= Sequential Organ Failure Assessment; SAPS II= simplified Acute Physiology Score; SAPS III= simplified Acute Physiology Score.

**Table S4-2: Correlation between the Phase Angle (°) and the disease severity**

| Study | N | Variables | Coefficient of correlation (r) | p value |
| --- | --- | --- | --- | --- |
| Berbigier 2013^[13]^ | 50 | SOFA | -0.005 | 0.97 |
|  |  | APACHE II | 0.210 | 0.16 |
| Paes 2018^[20]^ | 31 | SOFA | -0.277 | 0.14 |
|  |  | APACHE II | -0.579 | 0.0008 |
| Osuna-Padilla 2021^[32]^ | 67 | SOFA | −0.04 | 0.72 |
|  |  | APACHE II | −0.39 | 0.001 |

APACHE II= Acute Physiology and Chronic Health Evaluation; SOFA= Sequential Organ Failure Assessment

**Supplementary material 5:**

**Evaluation of the value of PA applications in nutrition among the included studies**

| Study | Objective | Nutritional assessment tool | Nutritional assessment associated results |
| --- | --- | --- | --- |
| Jansen 2019[^15]^ | Using PA in identifying malnutrition | Subjective Global Assessment | The accuracy of standardized PA (SPA) reduced in identifying malnourished patients was 60.6% (ROC curve AUC=0.606, 95% CI 0.519-0.694). |
| Kuchnia 2016^[17]^ | Using PA to assess low muscularity | Skeletal muscle cross-sectional area | Using linear regression, PA alone was able to predict 20% of the variance in CT muscle cross-sectional area and 61% of the variance when covariates were added to the model. |
| Lee 2015^[16]^ | Using PA to evaluate nutritional status | Serum albumin level and total lymphocyte count | Phase angle was significantly associated with the severity of nutritional status, with 4.5^o^±1.4^o^, 4.1^o^±1.1^o^, and 3.1^o^ ± 0.9^o^ in the well-nourished, moderately malnourished, and severely malnourished group patients, respectively. |
| Paes 2018^[20]^ | To evaluate the relationship between PA and nutritional status | The nutritional risk in the critically ill score | The median PA was significantly lower in patients with high nutritional risk assessed by the NUTRIC scores (high Risk: OR 2.7^o^ [IQR 2.1-3.9] vs. low Risk: OR 4.3^o^ [IQR 3.5-5.4]). |
| Razzera 2019^[19]^ | To evaluate the validity of PA as predictor of nutrition risk | The nutritional risk in the critically ill score | A PA <5.5^o^ showed an accuracy of 79% (95% CI 0.59-0.83) in identifying patients at high nutrition risk. |
| Buter 2017^[29]^ | Using PA to evaluate nutritional status | Short Nutritional Assessment Questionnaire | Phase angle was significantly higher in patients with a SNAQ score of 0-1 (5.5^o^±1.2 ^o^) than patients with a SNAQ score 2 (4.4^o^±1.1^o^) (P< 0.001). |
| Visser 2012^[33]^ | To evaluate the validity of PA as predictor of nutrition risk | Fat free mass index, BMI | From those patients with a low PA, 22.9% had a low fat free mass index while this was 1.3% in patients with a high PA (p <0.001) . Meanwhile, patients with a low PA had more often a low BMI (10.4% vs. 0.9%, p <0.001). |
| Yasui-Yamada 2020^[30]^ | Using PA to evaluate nutritional status | Subjective Global Assessment | The low-PA group showed a high prevalence of malnutrition (48%) than normal-PA (25%), and high-PA (9%) (P<0.001) |

**Supplementary material 6**

**Table S5 Quality assessment and overall risk of bias of included studies**

| First author / year | Patient selection | | | | Comparability | Outcome | | | Risk of bias |
| --- | --- | --- | --- | --- | --- | --- | --- | --- | --- |
|  | Representation of the exposed cohort | Selection of the non-exposed cohort | **Ascertainment of exposure** | **Outcome of**  **interest not**  **present at start** | **Comparability of cohorts on the basis of the design or analysis** | **Assessment**  **of outcome** | **Was follow-up long enough for outcomes to occur** | **Adequacy of follow up of cohorts** |  |
| Visser 2012^[33]^ | ★ | ★ | ★ | ☆ | ★★ | ★ | ★ | ★ | 8 |
| Berbigier 2013^[13]^ | ★ | ★ | ★ | ☆ | ★★ | ★ | ★ | ★ | 8 |
| Silva 2015^[28]^ | ★ | ★ | ★ | ☆ | ★★ | ★ | ★ | ★ | 8 |
| Lee 2015^[16]^ | ★ | ★ | ★ | ☆ | ★★ | ★ | ★ | ★ | 8 |
| Vermeulen 2016^[21]^ | ★ | ★ | ★ | ☆ | ★ | ★ | ★ | ★ | 7 |
| Thibault 2016^[18]^ | ★ | ★ | ★ | ☆ | ★★ | ★ | ★ | ★ | 8 |
| Kuchnia 2016^[17]^ | ★ | ★ | ★ | ☆ | ★★ | ★ | ★ | ★ | 8 |
| Stapel 2017^[34]^ | ★ | ★ | ★ | ☆ | ★ | ★ | ★ | ★ | 7 |
| Lee 2017^[14]^ | ★ | ★ | ★ | ☆ | ★★ | ★ | ★ | ★ | 8 |
| Buter 2017^[29]^ | ★ | ★ | ★ | ☆ | ★★ | ★ | ★ | ★ | 8 |
| Ellegard 2018^[22]^ | ★ | ★ | ★ | ☆ | ★★ | ★ | ★ | ★ | 8 |
| Paes 2018^[20]^ | ★ | ★ | ★ | ☆ | ★★ | ★ | ★ | ★ | 8 |
| Razzera 2019^[19]^ | ★ | ★ | ★ | ☆ | ★★ | ★ | ★ | ★ | 8 |
| Jansen 2019^[15]^ | ★ | ★ | ★ | ☆ | ★★ | ★ | ★ | ★ | 8 |
| Yao 2019^[23]^ | ★ | ★ | ★ | ☆ | ★★ | ★ | ★ | ★ | 8 |
| Yasui-Yamada 2020^[30]^ | ★ | ★ | ★ | ☆ | ★★ | ★ | ★ | ★ | 8 |
| Osuna-Padilla 2021^[32]^ | ★ | ★ | ★ | ☆ | ★★ | ★ | ★ | ★ | 8 |
| Ko 2020^[27]^ | ★ | ★ | ★ | ☆ | ★★ | ★ | ★ | ★ | 8 |
| Passos 2021^[31]^ | ★ | ★ | ★ | ☆ | ★★ | ★ | ★ | ★ | 8 |
| Paolo 2022^[35]^ | ★ | ★ | ★ | ☆ | ★★ | ★ | ★ | ★ | 8 |

**Abbreviations:** H=high quality; M=moderate quality; L= low quality.

**Note:** A study was given a maximum of one point in each item within the patient selection and outcome domains and given a maximum of two points for the Comparability domain with the following criteria:

1. **Representation of the exposed cohort**：Studies received 1 point if they recruited consecutive series of adult patients with PA values tested, or all included patients or did not miss a large number of patients.

2. **Selection of the non-exposed cohort**：Studies received 1 point if both groups of patients with or without reduced PA (defined by each author) were recruited from the same cohort.

3. **Ascertainment of exposure**: Studies received 1 point if they had been demonstrated to have reduced PA levels.

4. **Outcome of interest was not present at start of study**: Studies received points if they demonstrated the outcome of interest was not present at the start of the study.

5. **Comparability:** Studies received points if they controlled the age and gender (1 point); or any additional important factors such as disease severity (i.e., SOFA, SAPS3, ISS or APACHEII scores) or ethnicities, comorbidities, or there were no significant differences between reduced PA and normal PA level (1 point).

6. **Assessment of outcome**: Studies received 1 point if they had independent blind assessment or record linkage.

7. **Was follow-up long enough for outcomes to occur**: Studies received 1 point if they follow up until at least either inpatient mortality or for 30 days or had adequate record linkage.

8. **Adequacy of follow up for cohorts**: Studies received 1 point if all recruited subjects were all followed up, or the number lost to follow-up was unlikely to introduce bias (≤10%).

**Supplementary material 7: Publication bias**


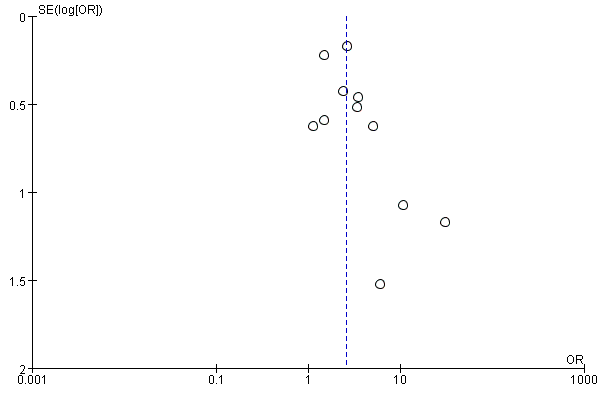


**Figure S6-1: Mortality between groups, 11 studies**


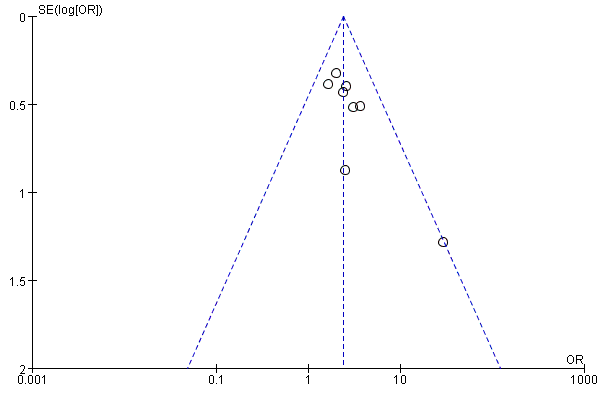


**Figure S6-2: Reduced PA to predict mortality(as categorical variable); 8 studies**


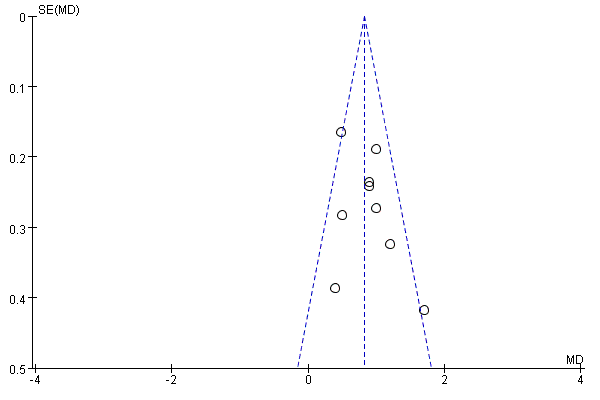


**Figure S6-3: PA between Survival and non-survival; 9 studies**


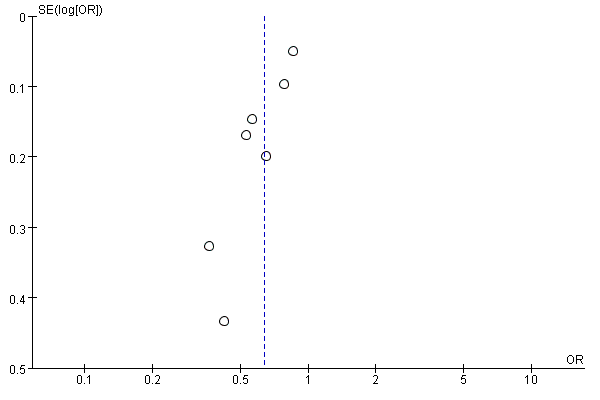


**Figure S6-4: PA to predict mortality (as continuous variable); 7 studies**
